# A Comparative Study of Black and White *Allium sativum* L.: Nutritional Composition and Bioactive Properties

**DOI:** 10.3390/molecules24112194

**Published:** 2019-06-11

**Authors:** Joana Botas, Ângela Fernandes, Lillian Barros, Maria José Alves, Ana Maria Carvalho, Isabel C.F.R. Ferreira

**Affiliations:** Centro de Investigação de Montanha (CIMO), Instituto Politécnico de Bragança, Campus de Santa Apolónia, 5300-253 Bragança, Portugal; joanacsbotas@gmail.com (J.B.); afeitor@ipb.pt (A.F.); lillian@ipb.pt (L.B.); maria.alves@ipb.pt (M.J.A.); anacarv@ipb.pt (A.M.C.)

**Keywords:** *Allium sativum* L., black garlic, commercial/traditionally cultivated, nutritional value, bioactive properties

## Abstract

Garlic (*Allium sativum* L.) has been used worldwide not only for its being a subject of dietary interest, but also for medicinal purposes, in prophylaxis, and for the treatment of diverse pathologies. New processing techniques have been developed and placed on the market in recent years to improve the organoleptic and nutritional value of food products. The present work aimed to study bulbils (cloves) of white (commercial and traditionally cultivated samples with different proveniences) and black (processed samples) garlic. All samples were compared with regard to their nutritional composition as well as their antioxidant and antimicrobial activities. Black garlic had the lowest moisture content but the highest total amount of sugars and energetic value. Black garlic also presented the highest antioxidant and antimicrobial (especially against methicillin-resistant *Staphylococcus aureus*) activities. Thus, black garlic, obtained by processing techniques, can be considered a promising product with high value that will be able to be exploited by the functional food/nutraceutical industry.

## 1. Introduction

*Allium sativum* L. (garlic) is a bulbous herbaceous plant that belongs to the Amaryllidaceae family. Garlic is originally from Asia [1] but is nonetheless now widely distributed around the world, with a cultivation area of 1,547,381 ha and an annual production of 25 million tonnes according to data regarding the year 2014 [2].

Garlic has been largely used since ancient times for dietary purposes but due to its medicinal applicability has also been used in the treatment of several diseases and prophylaxis. With regard to its medicinal properties, garlic has been described as being antimicrobial, antiseptic, antiviral, antioxidant, anticancer, immunostimulatory, cardioprotective, and hypoglycemic [1,3,4,5,6]. These properties are related to garlic’s bioactive molecules such as its organosulfur compounds [7], among which can be found allines (alkylcysteine sulfoxides) and non-volatile amino acids (thiosulfinates) [3,7,8]. These bioactive molecules also include fructosan (about 75%), reducing sugars (15%), thiocyanates (allyl thiocyanate and other allyl derivatives), minerals, saponins, and traces of vitamins (A, complex B, and C) [3]. 

Although garlic is generally associated with the improvement of health, there have been reports of its adverse effects when used in high doses, including allergic reactions, stomach problems, and diarrhea [8,9]. There are several formulations of garlic on the market in addition to fresh bulbous garlic, including extracts, essential oils, capsules, and garlic powder. These formulations show considerable variation in their chemical composition and bioactive properties [10,11]. For instance, garlic powder, when compared to fresh garlic, does not show hypoglycemic effects [11] and presents a lower antimicrobial activity [7]. Moreover, the geographic location and genotypes of garlic also have a strong influence on its chemical composition and bioactive properties. A previous study performed by Beato et al. [12] has compared *A. sativum* samples from different locations and genotypes, revealing that these two factors significantly influence garlic chemical composition. 

In past years new garlic products have emerged on the market which are more appealing to consumers than fresh garlic in that they enhance garlic’s scent and flavor. In order to develop these products, different techniques, such as aging, fermentation, and heat treatment, have been applied. Heat treatment consists of submitting garlic bulbs to controlled temperature and humidity conditions for a period of one month, with the product obtained within this process commonly known as black garlic. Black garlic presents different organoleptic characteristics, such as color (turns black), texture, flavor (sweeter), and odor (more pleasant) [13,14]. Choi et al. [14] have suggested that 70 °C and 90% relative humidity for 21 days are the optimal conditions for black garlic production; in addition to the organoleptic changes, heat treatment also causes chemical changes. However, several studies have stated that this process does not reduce garlic bioactive properties and indeed sometimes shows an enhancement of these properties [13,14,15,16].

There is a need to develop research around the conservation and exploitation of traditional diversity of local and commercial crops, this being an essential component for sustainable agriculture. In this sense, the present study compares the nutritional composition and bioactive properties of black garlic (garlic which has been submitted to heat treatment) and white garlic (unprocessed and with different proveniences) in order to valorize this product as a valuable processing system for garlic.

## 2. Results and Discussion

Results regarding the nutritional value and hydrophilic compounds of the *A. sativum* samples are presented in Table 1. 

Carbohydrates were the most abundant macronutrients, immediately followed by proteins. The highest values of carbohydrate and ash content were found in the black garlic sample (BG) in addition to the highest energetic contribution.

Protein content was higher in the traditional cultivated white garlic from Algarve (WGT-A), while fat content was higher both in the traditional cultivated white garlic from Trás-os-Montes (WGT-TM) and in BG, without significant statistical differences. Regarding fat and protein content, previous studies on white garlic samples have reported similar results as those found for white garlic cultivated in intensive farming systems (WGI). Nevertheless, lower ash content in comparison to the present study has been defined. [17,18,19]. 

The moisture content was higher in the WGT-TM sample and the lowest level was obtained for the BG sample. Other studies have also shown lower moisture percentages (about or less than 20%) for BG samples when compared to fresh white garlic samples [13,14]. The decrease in moisture content may be the result of the heat treatment conditions (temperature and humidity) that cause, among other things, changes in the bulbils’ texture. 

With regard to free sugars, fructose was the most abundant sugar in BG and sucrose was the most abundant in WGI, WGT-A, and WGT-TM. Sucrose was found in higher concentrations in WGT-A and in lower levels in BG. Xylose was only detected in BG. The highest content of glucose and fructose was found in BG and the lowest content was observed for WGT-A. Jeong et al. [15] have not detected glucose in BG, contrary to the findings of the present study, but their fresh white garlic samples presented a similar concentration to that of the WGI samples studied here. Fructose was also significantly higher in BG than in the fresh white garlic samples. Li et al. [20] and Mashayekhi et al. [21] have suggested that temperature and storage period decrease garlic sugars’ composition. During the sprouting process, sucrose, glucose, and fructose concentrations oscillate according to the role that each free sugar plays in bud growth [21].

Five organic acids were detected: oxalic, malic, pyruvic, citric, and fumaric acid. WGI had the highest content of total organic acids while BG had the lowest. For WGI, pyruvic acid was the predominant organic acid, followed by citric acid and oxalic acid. For WGT-A, the most abundant acid was pyruvic acid, while for WGT-TM it was citric acid. The highest acid concentration for BG was for malic acid, followed by oxalic acid; BG presented only trace levels of pyruvic acid. Fumaric acid was present in trace levels in all the samples, with the exception of BG. 

Twenty-seven fatty acids were identified (Table 2), of which saturated fatty acids (SFA) were predominant in BG, WGI, and WGT-TM, while for WGT-A, polyunsaturated fatty acids (PUFA) were predominant. PUFA predominated in relation to monounsaturated fatty acids (MUFA) in all samples, with the exception of BG. The most abundant fatty acids in BG were palmitic (C16:0), oleic (C18:1n9), and stearic (C18:0). For the remaining samples the most abundant fatty acids were linoleic (C18:2n6c), palmitic (C16:0), and oleic (C18:1n9). Capric (C10:0), lauric (C12:0), and palmitic (C16:0) acids have been previously identified in white garlic samples, but in lower percentages than in the present study [22].

α-Tocopherol was the only vitamer of tocopherols which was found in all samples and was predominant in WGI samples (Table 2).

Antioxidant activity was tested using four different methods, as shown in Table 3. All samples presented antioxidant activity, with BG being the sample that revealed the lowest extract concentration that provide 50% of antioxidant activity (EC_50_) values across all the assayed methods, which meant that this sample presented the highest antioxidant activity. 

With regard to the white garlic samples, WGI gave the highest DPPH scavenging activity, reducing power, and lipid peroxidation inhibition when evaluated by the thiobarbituric acid reactive substances (TBARS) assay, while WGT-TM presented the highest β-carotene bleaching inhibition. WGI, WGT-A, and WGT-TM showed little variation in EC_50_ values, while BG showed significantly lower values. Bae et al. [13], Choi et al. [14], and Jeong et al. [15] have described similar results for DPPH scavenging activity and reducing power using samples of fresh garlic purchased in Korean local markets and subjected to heat treatment by the authors. The white garlic samples used in this work also had similar DPPH scavenging activity and reducing power to those reported by Khalid et al. [18], who studied samples of white garlic from local markets in Pakistan.

Results regarding antibacterial activity evaluated towards pathogenic bacteria is presented in Table 4. The results were dose-dependent and varied according to the bacteria studied. The most sensitive bacterium was methicillin-resistant *Staphylococcus aureus* (MRSA) and the most resistant was *Klebsiella pneumoniae.* BG showed antibacterial activity against all the clinical isolates tested, of which the best minimal inhibitory concentration (MIC) results were obtained for the Gram-positive bacteria methicillin-susceptible *Staphylococcus aureus* (MSSA) and MRSA, followed by *Enterococcus faecalis* and *Listeria monocytogenes*. Regarding Gram-negative bacteria, the best MIC results were obtained for *Escherichia coli*, followed by *Pseudomonas aeruginosa.*

Regarding bactericidal action, the best minimal bactericidal concentration (MBC) value for BG was obtained for MRSA and *E. coli*, followed by MSSA, *P. aeruginosa*, and *Acinetobacter baumannii*. These microorganisms were isolated from infections related to healthcare and are a serious public health problem, meaning BG, considered as having great antimicrobial potential, could be used against these bacteria.

The white garlic samples (WGI, WGT-A, and WGT-TM) showed similar results for most of the studied bacteria and did not present bactericidal activity. However, WGT-TM presented the best inhibitory results with MIC values of 50 mg/mL against MRSA and 100 mg/mL against *L. monocytogenes,* MSSA, and *A. baumannii.* WGI showed inhibitory activity only against MRSA and WGT-A produced its best MIC values for *L. monocytogenes* and *A. baumanni.*

A previous study using white garlic extracts has also shown low antimicrobial activity against most of the studied strains, thus showing a higher inhibition against *S. aureus* [18]. Nevertheless, it should be mentioned that the strains studied herein were clinical isolates which present a high resistance to the majority of antibiotics. The results obtained are therefore very promising. 

## 3. Materials and Methods

### 3.1. Standards and Reagents

Acetonitrile 99.9%, *n*-hexane 95%, and ethyl acetate 99.8% of HPLC grade were obtained from Lab-Scan (Lisbon, Portugal). Methanol and all other chemicals were of analytical grade and were obtained from common sources. 

The fatty acids methyl ester (FAME) reference standard mixture 37 (standard 47885-U) was purchased from Sigma-Aldrich (St. Louis, MO, USA), as also were sugar standards, organic acids, and 6-hydroxy-2,5,7,8-tetramethylchroman-2-carboxylic acid (Trolox). Tocol and tocopherol standards were purchased from Matreya (Pleasant Gap, PA, USA). 

2,2-Diphenyl-1-picrylhydrazyl was obtained from Alfa Aesar (Ward Hill, MA, USA). *p*-iodonitrotetrazolic chloride (INT) dye was purchased from Sigma-Aldrich (St. Louis, MO, USA) and used as an indicator of microbial growth. Water was treated in a Milli-Q water purification system (TGI Pure Water Systems, Greenville, CA, USA).

### 3.2. Samples

The samples of *Allium sativum* L. (garlic) which were studied were produced as follows:

BG—black garlic samples were submitted to a heat treatment process by a Portuguese company.
WGI—white garlic samples were cultivated within an intensive farming system and obtained in 2015 in a large commercial area in the south of Spain.
WGT-A and WGT-TM—white garlic samples were cultivated within traditional farming systems and obtained in 2015 directly from the producers in local markets at Bragança (Trás-os-Montes, Portugal) and Silves (Algarve, Portugal), respectively.

All samples correspond to unidentified garlic varieties maintained by the producers.

The different garlic samples were lyophilized (FreeZone 4.5, Labconco, MO, USA) and ground to a fine powder (20 mesh) prior to analyses.

### 3.3. Nutritional Value and Hydrophilic Compounds

#### 3.3.1. Macronutrients and Energetic Value 

The samples were analyzed for their nutritional composition with regard to protein, fat, carbohydrates, and ash using the procedures of the Association of Official Agricultural Chemists (AOAC) [23]. 

Crude protein content (*N* × 6.25) was estimated via the macro-Kjeldahl method using an automatic distillation and titration unit (model Pro-Nitro-A, JP Selecta, Barcelona); crude fat was determined using a Soxhlet apparatus by extracting a known weight of powdered sample with petroleum ether; ash content was determined by incineration at 550 ± 15 °C. 

Total carbohydrates were calculated by difference and total energy was calculated according to the following equation:

energy (kcal) = 4 × (g _protein_ + g _carbohydrates_) + 9 × (g _fat_), (1)

#### 3.3.2. Free Sugars 

Free sugars were determined by high performance liquid chromatography coupled to a refraction index detector (HPLC-RI, Knauer, Smartline system 1000, Berlin, Germany) after extraction and analysis procedures previously described by the authors [24]. 

Sugar identification was performed by comparing the relative retention times of sample peaks with standards. Quantification was based on the RI signal response of each standard using the internal standard (IS, in this case melezitose) method, with the results being expressed in g per 100 g of fresh weight (fw). 

#### 3.3.3. Organic Acids

Organic acids were determined following a procedure previously described by the authors [25]. The analysis was performed using a Shimadzu 20A series UFLC (Shimadzu Coperation, Kyoto, Japan). 

Detection was carried out in a diode array detector (DAD) using 215 nm as the preferred wavelength. The organic acids which were found were quantified by comparison of the area of their peaks recorded at 215 nm with calibration curves obtained from commercial standards of each compound. The results were expressed in g per 100 g of fw. 

### 3.4. Lipophilic Compounds 

#### 3.4.1. Fatty Acids

Fatty acids were determined after a transesterification procedure that has described previously by the authors [24]. The fatty acid profiles were analyzed with a DANI 1000 gas chromatographer (DANI 1000, Contone, Switzerland) with a flame ionization detector (FID). Separation was achieved with a Macherey-Nagel (Duren, Germany) column (50% cyanopropyl-methyl–50% phenylmethylpolysiloxane, 30 m × 0.32 mm ID × 0.25 μm *d*f). 

Fatty acid identification was achieved by comparing the relative retention times of FAME peaks from samples with standards. The results were recorded and processed using Clarity 4.0.1.7 Software (DataApex, Podohradska, Czech Republic) and expressed in terms of the relative percentage of each fatty acid.

#### 3.4.2. Tocopherols

Tocopherols were determined as previously described by the authors Heleno et al. [26] by using an HPLC-fluorescence detector. The compounds were identified by chromatographic comparisons with authentic standards. 

Quantification was based on fluorescence signal response using the internal standard method (tocol) and tocopherol content was further expressed in µg per 100 g of fw.

### 3.5. Evaluation of Selected Bioactive Properties

#### 3.5.1. Extraction Procedure

The lyophilized powder (1.5 g) was stirred with methanol (30 mL) at 25 °C at 150 rpm for 1 h and then filtered through Whatman No. 4 paper. The residue was then extracted with an additional portion of methanol. The combined methanolic extracts were evaporated under reduced pressure (rotary evaporator Büchi R-210, Flawil, Switzerland), re-dissolved in methanol at 40 mg/mL (stock solution), and stored at 4 °C for further use.

For the antioxidant activity, successive dilutions were made from the stock solution as previously described by Sarmento et al. [27]. Sample concentrations providing 50% of antioxidant activity or 0.5 of absorbance were calculated from graphs of antioxidant activity percentages (DPPH, β-carotene/linoleate, and TBARS assays) or absorbance at 690 nm (reducing power assay) against sample concentrations.

Trolox was used as a standard. For the antimicrobial activity the extracts were dissolved in medium broth before inoculation with the bacteria and successive dilution were performed in the microplate.

#### 3.5.2. Antioxidant Activity 

*DPPH radical scavenging activity*. This methodology was performed using an ELX800 Microplate Reader (Bio-Tek, Winooski, VT, USA). The radical scavenging activity (RSA) was calculated as a percentage of DPPH discoloration using the equation % RSA = [(A_DPPH_ - A_S_)/A_DPPH_] × 100, where A_S_ is the absorbance of the solution when the sample extract has been added at a particular level and A_DPPH_ is the absorbance of the DPPH solution. 

*Reducing power*. This methodology was performed using the microplate reader described above and the capacity to convert Fe^3+^ into Fe^2+^ was evaluated by reading the absorbance at 690 nm.

*Inhibition of β-carotene bleaching*. This was evaluated though β-carotene/linoleate assays; the neutralization of linoleate free radicals, which avoids β-carotene bleaching, was calculated using the following equation: (absorbance after 2 h of assay/initial absorbance) × 100. 

*TBARS assay*. Lipid peroxidation inhibition in porcine brain homogenates was evaluated by observing decreases in TBARS; the color intensity of the malondialdehyde-thiobarbituric acid (MDA-TBA) was measured by its absorbance at 532 nm. The inhibition ratio (%) was calculated using the equation inhibition ratio (%) = [(A − B)/A] × 100%, where A and B are the absorbance of the control and the sample solution, respectively.

#### 3.5.3. Antimicrobial Activity

Antimicrobial activity screening was assessed according to Pereira et al. [28] using microorganisms obtained from clinical isolates (Hospital Center of Trás-os-Montes and Alto Douro, Vila Real, Portugal). 

Four Gram-negative bacteria (*E. coli*, isolated from urine; *P. aeruginosa*, isolated from expectoration; *A. baumannii,* isolated from expectoration; and *K. pneumonia,* isolated from urine) and four Gram-positive bacteria (MRSA, isolated from expectoration; MSSA, isolated from wound exudates; *E. faecalis*, isolated from urine; and *L. monocytogenes*, isolated from cerebrospinal fluid) were used to screen the antimicrobial activity of the garlic extracts. 

Microorganism identification and susceptibility tests were performed using MicroScan panels (MicroScan^®^, Siemens Medical Solutions Diagnostic, West Sacramento, CA, USA) via the microdilution plate method. Determination of minimum inhibitory concentrations and minimum bactericidal concentrations was performed using the microdilution method and rapid *p*-iodonitrotetrazolium chloride colorimetric assays. 

Viable microorganisms reduced the yellow dye to a pink color. MIC was defined as the lowest extract concentration that prevented this change and exhibited inhibition of bacterial growth and MBC was defined as the lowest extract concentration that killed the bacteria. The antibiotic susceptibility profile of the bacteria is presented as Supplementary Material (Table S1). 

### 3.6. Statistical Analysis 

Three repetitions of the samples were used and triplicates for each concentration reading were carried out in all the assays. 

The results were expressed as mean values ± standard deviation (SD) and analyzed using ANOVA, followed by Tukey’s Honestly Significant Difference (HSD) test with *p* = 0.05. Analyses were carried out using IBM SPSS Statistics for Windows, version 23.0 (IBM Corp., Armonk, New York, USA).

## 4. Conclusions

Overall, as has been previously described in the literature and demonstrated in the present study, one of the major beneficial effects of garlic is that related to its antioxidant properties and antimicrobial properties. Several authors have reported that processing treatments may alter chemical composition and therefore significantly affect antimicrobial and antioxidant properties of garlic products, mostly due to the labile nature of organosulfuric compounds. These significant differences in chemical composition and antioxidant activity could be attributed to adaptation mechanisms that may have developed throughout their cultivation history as well as to artificial selection occurring through vegetative propagation and pre-harvest factors [29]. In general, BG was the sample with the most promising bioactivity results. White garlic samples showed a slight variation in results, from which WGI stands out. Heat treatment (used for BG) was the factor that most influenced changes in chemical composition and bioactive properties.

Origin and cultivation methods have also shown some influence in the chemical and bioactive properties of garlic. To better evaluate this influence, it would be interesting to develop a study in which the cultivation conditions were controlled. From these results, it can also be concluded that innovative products, such as black garlic, can exhibit improved bioactive properties. It will therefore be of interest to study black garlic in more detail to better exploit the potential of this product.

## Figures and Tables

**Table 1:** Nutritional value and hydrophilic compounds of *A. sativum* samples (mean ± SD).

	BG	WGI	WGT-A	WGT-ATM
**Moisture (%)**	54 ± 3 ^c^	62 ± 7 ^b^	64.7 ± 0.5 ^b^	74 ± 2 ^a^
**Nutritional value**		**g/100 g fw**		
Fat	0.722 ± 0.001 ^a^	0.47 ± 0.02 ^c^	0.67 ± 0.03 ^b^	0.74 ± 0.02 ^a^
Proteins	7.4 ± 0.1 ^b^	6.5 ± 0.1 ^c^	7.8 ± 0.2 ^a^	5.2 ± 0.1 ^d^
Ash	3.2 ± 0.1 ^a^	2.9 ± 0.1 ^b^	2.7 ± 0.2 ^c^	1.60 ± 0.04 ^d^
Carbohydrates	35 ± 3 ^a^	28 ± 2 ^b^	24.2 ± 0.4 ^b^	18 ± 2 ^c^
Energy (kcal/100 g fw)	177 ± 8 a	141 ± 8 ^b^	134 ± 2 ^b^	100 ± 9 ^c^
**Free sugars**		**g/100 g fw**		
Xylose	0.82 ± 0.01	nd	nd	nd
Fructose	30.4 ± 0.7 ^a^	0.45 ± 0.01 ^b^	0.09 ± 0.01 ^d^	0.20 ± 0.01 ^c^
Glucose	2.14 ± 0.03 ^a^	0.28 ± 0.02 ^b^	0.04 ± 0.01 ^d^	0.12 ± 0.01 ^c^
Sucrose	0.23 ± 0.05 ^d^	0.58 ± 0.01 ^b^	1.35 ± 0.01 ^a^	0.38 ± 0.01 ^c^
Total	33.6 ± 0.7 ^a^	1.32 ± 0.05 ^bc^	1.48 ± 0.01 ^b^	0.70 ± 0.01 ^c^
**Organic acids**		**g/100 g fw**		
Oxalic acid	0.12 ± 0.02 ^c^	0.13 ± 0.01 ^c^	0.257 ± 0.001 ^a^	0.20 ± 0.01 ^b^
Malic acid	0.32 ± 0.01 ^a^	tr	0.006 ± 0.003 ^b^	0.32 ± 0.01 ^a^
Pyruvic acid	tr	1.43 ± 0.01 ^a^	1.38 ± 0.01 ^b^	0.10 ± 0.01 ^c^
Citric acid	nd	1.07 ± 0.01 ^a^	0.57 ± 0.02 ^c^	0.82 ± 0.02 ^b^
Fumaric acid	nd	tr	tr	tr
Total	0.430 ± 0.001 ^d^	2.64 ± 0.01 ^a^	2.21 ± 0.01 ^b^	1.44 ± 0.01 ^c^

Legend: fw, fresh weight basis; nd, not detected; tr, Traces; BG, black garlic; WGI, white garlic obtained by intensive farming in Spain; WGT-A, white garlic obtained by traditional farming at Algarve (Portugal); WGT-TM, white garlic obtained by traditional farming at Trás-os-Montes (Portugal). In each row different letters indicate significant differences (*p* < 0.05).

**Table 2:** Chemical composition with regard to lipophilic compounds of *A. sativum* samples (mean ± SD).

	BG	WGI	WGT-A	WGT-ATM
Fatty Acids		Relative Percentage (%)		
C6:0	1.4 ± 0.1	1.01 ± 0.01	0.61 ± 0.05	1.4 ± 0.1
C8:0	1.15 ± 0.06	0.75 ± 0.01	0.47 ± 0.03	1.05 ± 0.06
C10:0	3.06 ± 0.01	1.93 ± 0.04	1.17 ± 0.04	2.61 ± 0.06
C11:0	0.02 ± 0.01	0.01 ± 0.01	0.15 ± 0.01	0.01 ± 0.01
C12:0	1.80 ± 0.07	1.15 ± 0.04	0.69 ± 0.01	1.46 ± 0.02
C13:0	0.06 ± 0.01	0.04 ± 0.01	0.02 ± 0.01	0.05 ± 0.01
C14:0	6.7 ± 0.3	4.4 ± 0.2	2.59 ± 0.02	5.5 ± 0.3
C14:1	0.10 ± 0.01	0.07 ± 0.01	0.05 ± 0.01	0.09 ± 0.01
C15:0	1.00 ± 0.04	0.72 ± 0.03	0.55 ± 0.01	0.80 ± 0.02
C16:0	25.3 ± 0.5	20.9 ± 0.4	16.80 ± 0.03	21.8 ± 0.2
C16:1	0.64 ± 0.03	0.56 ± 0.01	0.49 ± 0.01	0.67 ± 0.01
C17:0	1.30 ± 0.02	1.08 ± 0.01	0.75 ± 0.01	1.02 ± 0.01
C18:0	16.4 ± 0.2	11.2 ± 0.1	7.13 ± 0.01	12.4 ± 0.2
C18:1n9	24.1 ± 0.3	18.4 ± 0.2	12.38 ± 0.04	20.4 ± 0.2
C18:2n6c	11.0 ± 0.1	29.2 ± 0.3	46.8 ± 0.1	24.0 ± 0.3
C18:3n3	2.14 ± 0.01	5.03 ± 0.04	5.74 ± 0.01	3.61 ± 0.03
C20:0	0.95 ± 0.03	0.80 ± 0.02	0.77 ± 0.07	0.77 ± 0.02
C20:1	0.05 ± 0.01	0.08 ± 0.01	0.08 ± 0.01	0.08 ± 0.01
C20:2	0.01 ± 0.01	0.04 ± 0.01	0.05 ± 0.01	0.04 ± 0.01
C20:3n6	0.06 ± 0.01	0.05 ± 0.01	0.02 ± 0.01	0.04 ± 0.01
C20:4n6	0.29 ± 0.01	0.17 ± 0.01	0.09 ± 0.01	0.21 ± 0.01
C20:3n3	0.31 ± 0.01	0.23 ± 0.01	0.22 ± 0.01	0.21 ± 0.01
C20:5n3	0.12 ± 0.01	0.05 ± 0.01	0.06 ± 0.01	0.12 ± 0.01
C22:0	1.18 ± 0.05	1.23 ± 0.05	1.35 ± 0.01	1.07 ± 0.04
C22:1n9	0.03 ± 0.01	0.09 ± 0.01	nd	0.04 ± 0.01
C23:0	0.23 ± 0.01	0.36 ± 0.02	0.37 ± 0.01	0.31 ± 0.02
C24:0	0.57 ± 0.06	0.52 ± 0.01	0.57 ± 0.04	0.41 ± 0.01
**SFA**	61.1 ± 0.4 ^a^	46.0 ± 0.5 ^c^	34.0 ± 0.2 ^d^	50.6 ± 0.5 ^b^
**MUFA**	25.0 ± 0.3 ^a^	19.2 ± 0.2 ^c^	13.00 ± 0.03 ^d^	21.2 ± 0.2 ^b^
**PUFA**	13.90 ± 0.09 ^d^	34.8 ± 0.3 ^b^	53.0 ± 0.1 ^a^	28.2 ± 0.3 ^c^
**Tocopherols**		**μg/100 g fw**		
α-Tocopherol	180 ± 2 ^b^	203 ± 4 ^a^	140 ± 1 ^d^	152 ± 3 ^c^

Legend: SFA, saturated fatty acids; MUFA, monounsaturated fatty acids; PUFA, polyunsaturated fatty acids. In each row different letters indicate significant differences (*p* < 0.05).

**Table 3:** Antioxidant properties of *A. sativum* samples (mean ± SD).

	BG	WGI	WGT-A	WGT-ATM
		EC_50_ values (mg/mL)	
DPPH scavenging activity	4.4 ± 0.2 ^d^	26 ± 1 ^c^	33.8 ± 0.2 ^a^	31 ± 1 ^b^
Reducing power	1.25 ± 0.04 ^d^	14.7 ± 0.2 ^c^	26.9 ± 0.2 ^a^	19.9 ± 0.2 ^b^
β-carotene bleaching inhibition	0.27 ± 0.01 ^d^	0.44 ± 0.01 ^b^	0.46 ± 0.01 ^a^	0.39 ± 0.01 ^c^
TBARS inhibition	0.39 ± 0.01 ^d^	0.88 ± 0.06 ^c^	2.3 ± 0.1 ^a^	1.0 ± 0.1 ^b^

Legend: EC_50_, extract concentration corresponding to 50% of antioxidant activity or 0.5 of absorbance in reducing power assay; TBARS, thiobarbituric acid reactive substances. Trolox (positive control) EC_50_ values: 42 µg/mL (DPPH scavenging activity), 41 µg/mL (reducing power), 18 µg/mL (β-carotene bleaching inhibition), and 23 µg/mL (TBARS inhibition). In each row different letters mean significant differences (*p* < 0.05).

**Table 4:** Antimicrobial activity of *A. sativum* samples.

Bacterial Strains		Gram-Positive				Gram-Negative			
**Garlic Samples**		*Enterococcus* *faecalis* CHTMAD	*Listeria* *monocytogenes*	Methicillin-susceptible *Staphylococcus aureus*	Methicillin-resistant *Staphylococcus aureus*	*Acinetobacter baumannii*	*Escherichia* *coli*	*Klebsiella pneumoniae*	*Pseudomonas aeruginosa*
**BG**	**MIC** MBC	6.25 >100	12.5 >100	3.125 100	3.125 50	100 100	25 50	100 >100	50 100
**WGI**	**MIC** MBC	>100 >100	>100 >100	>100 >100	100 >100	>100 >100	>100 >100	>100 >100	>100 >100
**WGT-A**	**MIC** MBC	>100 >100	100 >100	>100 >100	>100 >100	100 >100	>100 >100	>100 >100	>100 >100
**WGT-ATM**	**MIC** MBC	>100 >100	100 >100	100 >100	50 >100	100 100	>100 >100	>100 >100	>100 >100

Legend: CHTMAD, Centro Hospitalar de Trás-os-Montes e Alto Douro; MIC, minimal inhibitory concentration; MBC, minimal bactericidal concentration. Both MIC and MBC are expressed in mg/mL.

## Supplementary Materials

The following are available online, Table S1: Resistance profile of Gram-positive and Gram-negative bacteria to different antibiotics; MIC values (µg/mL).
