# Supplementary material for: A Comparative Study of Black and White Allium sativum L.: Nutritional Composition and Bioactive Properties
Source: Molecules. 2019 Jun 11;24(11):2194. doi: 10.3390/molecules24112194 (PMC6600231; doi:10.3390/molecules24112194)
Supplement: Supplementary file 1 [file molecules-24-02194-s001.pdf]

Supplementary material.

Table S1. Resistance profile of Gram-positive and Gram-negative bacteria to different antibiotics; MIC values (µg/mL).

	Amoxicillin/Clavulanic Acid	Amikacin	Ampicillin	Gentamicin	Tobramycin	Vancomycin
**Gram-negative**						
E. coli	≤8/4<br>S	na	>16<br>R	≤2<br>R	na	
K. pneumoniae	≤8/4<br>S	na	>16<br>R	≤2<br>S	na	
A. baumannii	na	na	na	>4<br>R	<2<br>S	
P. aeruginosa	na	≤8<br>S	na	> 4<br>R	> 4<br>R	
**Gram-positive**						
MRSA	na	na	na	na	na	≤2<br>S
MSSA	na	na	na	na	na	≤2<br>S
E. faecalis	na	na	≤1<br>S	na	na	≤2<br>S
L. monocytogenes	na	na	≤1<br>S	na	na	na

MSSA - methicillin-sensitive *Staphylococcus aureus*; MRSA - methicillin-resistant *Staphylococcus aureus*; S- Susceptible; ; R- Resistant: this classification was made according to the interpretative breakpoints suggested by Clinical and Laboratory Standards Institute (CLSI) and European Committee on Antimicrobial Susceptibility Testing (EUCAST); na- not applicable.
